# Impact of hs-CRP concentration on brain structure alterations and cognitive trajectory in Alzheimer’s disease

**DOI:** 10.3389/fnagi.2023.1227325

**Published:** 2023-08-01

**Authors:** Ye Zhang, Yasuko Tatewaki, Taizen Nakase, Yingxu Liu, Naoki Tomita, Benjamin Thyreau, Haixia Zheng, Michiho Muranaka, Yumi Takano, Tatsuo Nagasaka, Yasuyuki Taki

**Affiliations:** ^1^Department of Aging Research and Geriatric Medicine, Institute of Development, Aging and Cancer, Tohoku University, Sendai, Japan; ^2^Department of Geriatric Medicine and Neuroimaging, Tohoku University Hospital, Sendai, Japan; ^3^Smart-Aging Research Center, Tohoku University, Sendai, Japan; ^4^Laureate Institute for Brain Research, Tulsa, OK, United States; ^5^Division of Radiology, Tohoku University Hospital, Sendai, Japan

**Keywords:** gray matter volume, systemic inflammation, C-reactive protein, FreeSurfer, subjective cognitive decline, Alzheimer’s disease

## Abstract

**Introduction:**

Present study was to investigate hs-CRP concentration, brain structural alterations, and cognitive function in the context of AD [Subjective cognitive decline (SCD), mild cognitive impairment (MCI), and AD].

**Methods:**

We retrospectively included 313 patients (Mean age = 76.40 years, 59 SCD, 101 MCI, 153 AD) in a cross-sectional analysis and 91 patients (Mean age = 75.83 years, 12 SCD, 43 MCI, 36 AD) in a longitudinal analysis. Multivariable linear regression was conducted to investigate the relationship between hs-CRP concentration and brain structural alterations, and cognitive function, respectively.

**Results:**

Hs-CRP was positively associated with gray matter volume in the left fusiform (β = 0.16, *p*_FDR_ = 0.023) and the left parahippocampal gyrus (β = 0.16, *p*_FDR_ = 0.029). *Post hoc* analysis revealed that these associations were mainly driven by patients with MCI and AD. The interaction of diagnosis and CRP was significantly associated with annual cognitive changes (β = 0.43, *p* = 0.008). Among these patients with AD, lower baseline CRP was correlated with greater future cognitive decline (*r* = −0.41, *p* = 0.013).

**Conclusion:**

Our study suggests that increased hs-CRP level may exert protective effect on brain structure alterations and future cognitive changes among patients already with cognitive impairment.

## 1. Introduction

Mounting and compelling evidence suggest that systemic inflammatory process may play a central role in the development of Alzheimer’s disease (AD) (McGeer and McGeer, 1998; Kuo et al., 2005; Holmes, 2013; Heppner et al., 2015; Kinney et al., 2018). Inflammatory proteins [i.e., Interleukin-6 (IL-6) and C-reactive protein (CRP)] have been detected in brain plaques and neurofibrillary tangles in patients with AD (Iwamoto et al., 1994; Huell et al., 1995), suggesting the involvement of inflammatory processes in the etiology of AD. As an acute-phase reactant, CRP is a relatively stable but non-specific marker of systemic inflammation which is primarily regulated by IL-6 (Pepys and Hirschfield, 2003). Clinically, CRP concentration was associated with age-related diseases, and was used as a marker of cardiovascular risk (Verma et al., 2005). Notably, peripheral CRP levels was moderately correlated with other inflammatory cytokines such as IL-6, IL-1, tumor necrosis factor (TNF) (*r* = 0.232–0.537, all *p* < 0.05), and was associated with cerebrospinal fluid CRP (*r* = 0.855, *p* < 0.001), suggesting that peripheral CRP is a useful marker for both peripheral and central immune activity (Felger et al., 2020).

The progression of AD is mainly characterized by cognitive decline and brain atrophy (Kinney et al., 2018). Despite mounting of evidence suggest that increased CRP concentration may have a deleterious impact on cognitive function and future risk of AD on non-demented populations (Teunissen et al., 2003; Engelhart et al., 2004; Komulainen et al., 2007; Kravitz et al., 2009; Wersching et al., 2010), conflicting findings have been reported in patients diagnosed with AD dementia and patients with mild cognitive impairment (MCI) (Locascio et al., 2008; O’Bryant et al., 2010; Silverman et al., 2012; Fernandes et al., 2020; Lewis and Knight, 2021). For instance, Fernandes et al. (2020) found that lower CRP level predicted a faster conversion to future AD dementia in patients with mild cognitive impairment (MCI), suggesting that the impact of CRP on AD pathology may vary with the disease progression. Supporting this notion, in a recent 10-year longitudinal study, increased CRP levels has been linked to worse future cognitive function only among cognitively healthy older participants; while in patients with dementia, elevated CRP was associated with slower future cognitive decline (Lewis and Knight, 2021). This evidence raised the question of whether CRP concentration associates with different brain alterations patterns in the context of different disease progression. In fact, several neuroimaging studies attempted to explore these relationships. CRP concentration has been linked to greater white matter hyperintensities severity (a loss of brain vascular integrity due to axonal loss and demyelination) (Young et al., 2008; Satizabal et al., 2012; Walker et al., 2018), decreased fractional anisotropy (Wersching et al., 2010; Wooten et al., 2021), greater regional cerebral blood flow decline (Warren et al., 2018). Moreover, increased CRP concentration has been linked to smaller gray matter volumes (GMVs) in the medial temporal lobe and cingulate cortex (Bettcher et al., 2012; Taki et al., 2013; Marsland et al., 2015), which may explain the association between CRP level and decreased cognitive function in cognitively healthy older adults (Teunissen et al., 2003; Engelhart et al., 2004; Komulainen et al., 2007; Kravitz et al., 2009; Wersching et al., 2010). However, it should be noted that these findings were lacking replication in patients already with cognitive impairment. Moreover, these studies employed whole-brain exploratory analysis, which is limited in lacking specificity of AD and immune-related neuroanatomical changes. Furthermore, the relationship between CRP and AD risk may be largely confounded by cardiovascular disease (CVD), which is highly associated with peripheral CRP level (Koyama et al., 2013). In a previous study, participants with increased CRP concentration at baseline increased over 50% risk of developing dementia; while this association no longer remained significant after adjusting for possible confounding of CVD risk factors (Hsu et al., 2017). Therefore, a strict exclusion based on detailed medical history are needed to control possible confounding variables, such as CVD and other diseases.

The present study had two main goals: (1) to investigate whether CRP is associated with brain structural alterations in the context of AD [patients with subjective cognitive decline (SCD), patients with MCI and patients with AD], and (2), whether CRP is associated with cognitive changes among patients with different stages of cognitive impairments. Findings from present study may contribute to mechanistic understanding of association between systemic inflammation, cognitive changes, and brain structural alterations in AD.

## 2. Materials and methods

### 2.1. Participants

Approval for present study was acquired by Clinical Research Ethics Committee of the Tohoku University Graduate School of Medicine (approval number: 2018-1-618).

374 participants (Mean age = 76.41 years, female: 58%) from the memory clinic at Tohoku University Hospital were screened. Data was collected from January 2018 to April 2022 only among those participants who first visited our memory clinic. Exclusion criteria were cerebral stroke, systemic lupus erythematosus, Parkinson’s disease, progressive supranuclear palsy, depression, infections, encephalitis, alcohol-related brain damage, schizophrenia, AD with cardiovascular disease, and other non-AD dementias. Clinical diagnoses were determined by certificated gerontologists or neurologists (T.N., N.T., Y. Taka., and M.M.) based on a multidisciplinary diagnostic evaluation, including a structured clinical interview, a neurological examination, neuropsychological testing, blood tests, and neuroimaging (structural magnetic resonance imaging: structural MRI). Diagnosis for AD and MCI were based on the criteria of the National Institute on Aging-Alzheimer’s Association (NIA-AA) working group (McKhann et al., 2011; Jack et al., 2018). Both “possible” and “probable” AD were included. All patients with MCI were amnestic type. Diagnosis for SCD was determined when individuals complained about subjective cognitive decline while clinical assessments, including neuropsychological assessments and neuroimaging, but did not indicate any neurological or psychiatric disorders (Jessen et al., 2014).

Participants completed Japanese version of Mini Mental Status Examination (MMSE-J) and Alzheimer’s Disease Assessment Scale Cognitive Subscale Japanese version (ADAS-cog-J) for assessing global cognitive function. Geriatric Depression Scale (GDS) was measured to assess depressive scores. All neuropsychological tests were performed by a clinical psychologist. Drinking habits was assessed by asking the participants whether they have a habit to continuously drink more than 72 ml of conversion pure alcohol per day. Smoking habits was measured by asking the participants whether they currently have a habit of smoking cigarette. Systolic blood pressure (SBP) and diastolic blood pressure (DBP) were also obtained. Body mass index (BMI) was also calculated. All information were adopted from clinical records during the first visit.

### 2.2. C-reactive protein

Using blood sample at the first visit, serum concentration of high sensitivity c-reactive protein (hs-CRP) were analyzed with the N-assay LA CRP-U Nittobo Kit (Nittobo Medical Corporation, Japan) in duplicates using Canon TBA-FX8 (Canon Medical Systems Corporation, Japan) in our hospital laboratory. The lowest limit of quantification (LLOQ) was 0.005 mg/dL, and the upper limit of quantification (ULOQ) was 40 mg/dL. The coefficient of variation was under 5%. The hs-CRP concentration was log-transformed due to skewed distributions. Patients who showed CRP level higher than 3 mg/dL were excluded from this study, because these patients can be assumed to have acute infectious disease (*n* = 3).

### 2.3. MRI acquisition and processing

T1-weighted MRI were acquired using magnetization-prepared rapid gradient echo sequence (MPRAGE) on a 3-Tesla Philips Achieva scanner with a 32-channel head coil using the following parameters: repetition time = 8.70 ms; echo time = 3.1 ms; flip angle = 8 degrees; field of view = 256 × 256 × 180 mm; and voxel dimensions = 0.7 × 0.7 × 0.7 mm.

**FIGURE 1:**
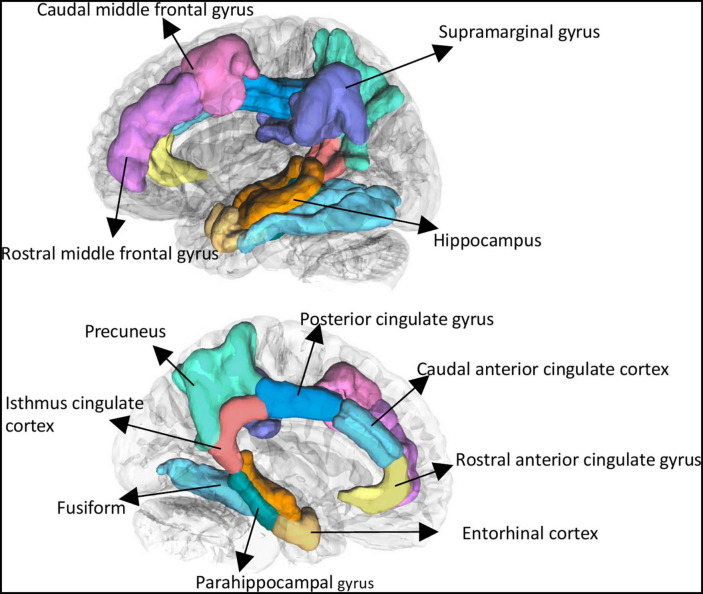
Selected brain regions of interest using Freesurfer. These regions included bilateral following regions: caudal anterior cingulate cortex, caudal middle frontal gyrus, entorhinal cortex, fusiform, isthmus cingulate cortex, parahippocampal gyrus, posterior cingulate cortex, precuneus, rostral anterior cingulate cortex, rostral middle frontal gyrus, supramarginal gyrus, and subcortical regions of hippocampus.

All imaging data were processed using FreeSurfer 7.0.0 (Fischl, 2012).^1^ Gray matter volume (GMVs) was extracted based on the Desikan–Killiany atlas, including 68 cortical regions (34 regions per hemisphere) (Desikan et al., 2006). We selected 24 regions of interest (ROIs) which were particularly vulnerable for AD progression (Baron et al., 2001; Sabuncu et al., 2012; Corlier et al., 2018) and systemic inflammation (Marsland et al., 2015; Corlier et al., 2018). These regions included bilateral following regions: caudal anterior cingulate cortex, caudal middle frontal gyrus, entorhinal cortex, fusiform, isthmus cingulate cortex, parahippocampal gyrus, posterior cingulate cortex, precuneus, rostral anterior cingulate cortex, rostral middle frontal gyrus, supramarginal gyrus and subcortical regions of hippocampus (see Figure 1). Estimate total intracranial volume (eTIV) were calculated. Quality check of the imaging was performed before analysis by a certificated neuroradiologist (Y. Tat).

### 2.4. Assessment of longitudinal cognitive changes

Then, we investigated the relationships between hs-CRP and follow-up cognitive changes in these patients. Among these 313 participants, we included 91 patients (mean age: 75.83 years, 12 SCD, 43 MCI, 36 AD) with longitudinal cognitive assessments. The follow-up neuropsychological assessment was ADAS-cog-J. ADAS-cog-J is a validated and structured scale often used to measure cognitive change in clinical practice (Raghavan et al., 2013). The ADAS-cog-J score ranges from 0 to 70, with higher scores indicates worse cognitive function. Due to a wide range of follow-up time (average follow-up time: 425 days, range: 96–902 days), the annual ADAS -cog change was calculated by using a formula: (the follow-up ADAS-cog-J scores - the baseline ADAS-cog-J scores)/the follow-up time (years). Greater positive annual ADAS-cog-J change indicates greater cognitive decline.

### 2.5. Statistical analyses

Statistical analyses were performed using RStudio and R version 4.1.2 (https://www.R-project.org/). For demographic information, data were presented as the mean ± SD for continuous variables and frequency and proportions for categorical variables. One-way analysis of variance was used to compare means. Additionally, Pearson correlation coefficients were calculated to illustrate the relationship between all demographic variables for all participants, SCD group, MCI group and AD group, separately (Supplementary Figures 1–4).

Multivariable robust linear regression models were conducted to investigate hs-CRP concentration on brain regions after adjusting for age, sex, MMSE-J, drinking habits, BMI, education years and eTIV. The false discovery rate (FDR) was set to 0.05 to control for multiple comparisons. Effect size estimates were calculated by standardized beta coefficient with 95% confidence interval. For brain regions survived after FDR correction, *post hoc* tests were performed to examine the effect of CRP concentration on these brain regions in each group, separately.

Then, we performed multivariable linear regression analysis to investigate the association between hs-CRP and cognitive function. Hs-CRP concentration was entered an independent variable, while baseline MMSE-J scores, baseline ADAS-cog-J scores and annual ADAS-cog-J changes were set as predictors, respectively. We included different three models: model 1 was unadjusted; model 2 was adjusted for age, sex, education, and diagnosis; and model 3 added the hs-CRP × diagnosis interaction variable. Then, we conducted *post-hoc* tests to examine the effect of CRP concentration on cognitive function in SCD, MCI, and AD.

## 3. Results

### 3.1. Study population

**FIGURE 2:**
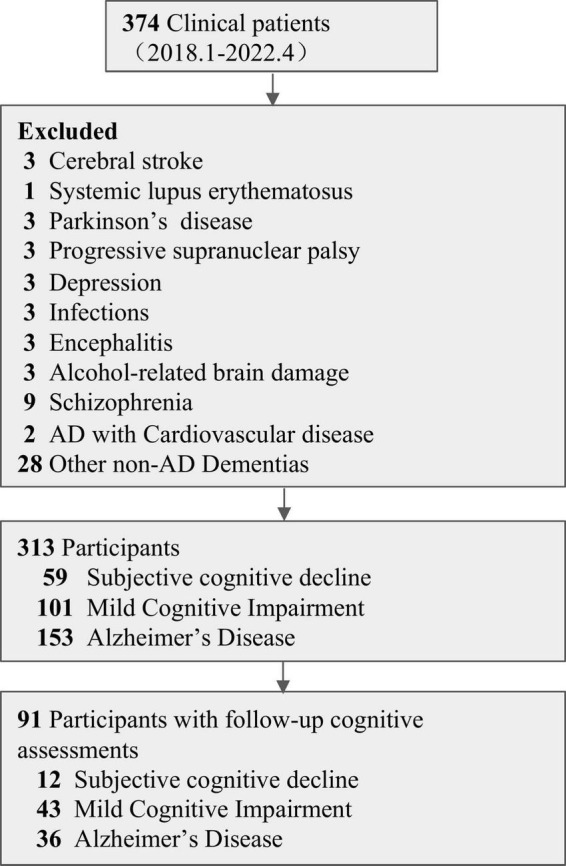
Data exclusion flow chart.

We excluded 61 patients, leaving a total of 313 patients (59 SCD, 101 MCI, 153 AD) were enrolled in present study (for data exclusion process, see Figure 2). As showed in the Table 1, for all participants, age, MMSE-J, BMI, ADAS-cog-J, and drinking habits showed statistically significant group difference. While there was not statistically significant group difference in sex, logCRP, SBP, DBP, GDS, smoking habits and eTIV. Among those participants with longitudinal ADAS scores, sex, MMSE, ADAS-cog baseline showed statistically significant group difference (Table 1).

**TABLE 1:** Demographic characteristics of study participants (*N* = 313).

	SCD		MCI		AD		*p^a^*
	*N*	Mean (SD)	*N*	Mean (SD)	*N*	Mean (SD)	
Age	59	68.81 (12.60)	101	76.39 (8.21)	153	79.35 (7.13)	0.001
Sex (Male,%)	59	25 (42.37%)	101	44 (43.56%)	153	56 (36.60%)	0.492
MMSE	58	28.28 (1.56)	101	24.59 (2.95)	153	19.86 (4.35)	0.000
BMI	59	23.62 (3.12)	101	22.41 (3.03)	153	22.37 (3.64)	0.034
logCRP	59	−1.24 (0.51)	101	−1.31 (0.49)	153	−1.21 (0.55)	0.494
Education years	59	13.46 (2.25)	101	12.81 (2.48)	153	12.40 (2.60)	0.012
SBP	57	134.05 (21.39)	99	140.91 (20.57)	149	139.68 (21.68)	0.181
DBP	57	76.63 (11.16)	98	72.65 (10.37)	148	73.89 (14.11)	0.316
ADAS-cog	52	6.27 (2.42)	97	12.10 (4.70)	143	19.19 (7.61)	0.001
GDS	41	4.22 (3.03)	78	3.54 (2.83)	102	3.68 (2.79)	0.550
Drinking habits (Yes,%)	59	41 (69.49%)	101	48 (47.52%)	153	74 (48.37%)	0.012
Smoking habits (Yes,%)	59	25 (42.37%)	101	34 (33.66%)	153	41 (27.80%)	0.084
eTIV	59	1544444.02 (185389.79)	101	1544625.15 (182104.28)	153	1517549.03 (159348.75)	0.216
Age	12	74.25 (12.2)	43	75.81 (8.36)	36	76.39 (7.14)	0.752
Sex (Male,%)	12	7 (58.33%)	43	21 (48.84%)	36	9 (25.00%)	0.041
MMSE	12	28.5 (1.24)	43	25.14 (2.53)	36	21.17 (3.96)	0.000
BMI	12	23.1 (3.32)	43	22.11 (2.95)	36	22.56 (4.33)	0.071
logCRP	12	−1.13 (0.58)	43	−1.30 (0.59)	36	−1.40 (0.48)	0.166
Education years	12	13.83 (1.75)	43	12.72 (2.66)	36	12.64 (2.4)	0.322
SBP	12	5.17 (2.92)	43	19.12 (10.2)	36	13.89 (7.04)	0.310
DBP	12	5.83 (3.21)	43	10.84 (6.52)	36	14.44 (7.26)	0.106
ADAS-cog baseline	12	6.53 (2.01)	43	11.25 (4.41)	36	17.02 (6.56)	0.001
Annual ADAS-cog change	12	0.66 (2.84)	43	0.30 (4.21)	36	2.42 (4.65)	0.083
GDS	12	3.92 (2.11)	43	5.95 (3.27)	36	6.33 (3.14)	0.657
Drinking habits (Yes,%)	12	8 (66.67%)	43	24 (55.81%)	36	18 (50.00)	0.596
Smoking habits (Yes,%)	12	4 (33.33%)	43	17 (39.53%)	36	10 (27.78%)	0.546
eTIV	12	1548775.7 (198378.54)	43	1551422.68 (184160)	36	1472160.93 (159649.25)	0.122

SCD, subjective cognitive decline; MCI, mild cognitive impairment; AD, Alzheimer’s disease; MMSE, Mini-Mental State Examination; BMI, body mass index; logCRP, CRP concentration was log-transformed; SBP, systolic blood pressure; DBP, diastolic blood pressure; ADAS-cog, Alzheimer’s Disease Assessment Scale-Cognitive subscale Japanese version; GDS, The Geriatric Depression Scale; eTIV: estimated Total Intracranial Volume. Drinking habits was assessed by asking the participants whether they have a habit to drinking alcohol. Smoking habits was measured by asking the participants whether they have a habit of smoking cigarette.

^a^Calculated using one-way analysis of variance for normally distributed continuous variables (Age, BMI, logCRP, SBP, DBP, ADAS-cog, ADAS-cog baseline, annual ADAS-cog change and eTIV), Kruskal-Wallis test for non-normally distributed continuous variables (MMSE, Education, GDS) and the χ^2^ test for categorical variables (Sex, drinking habits and smoking habits).

### 3.2. Gray matter volume and hs-CRP

**FIGURE 3:**
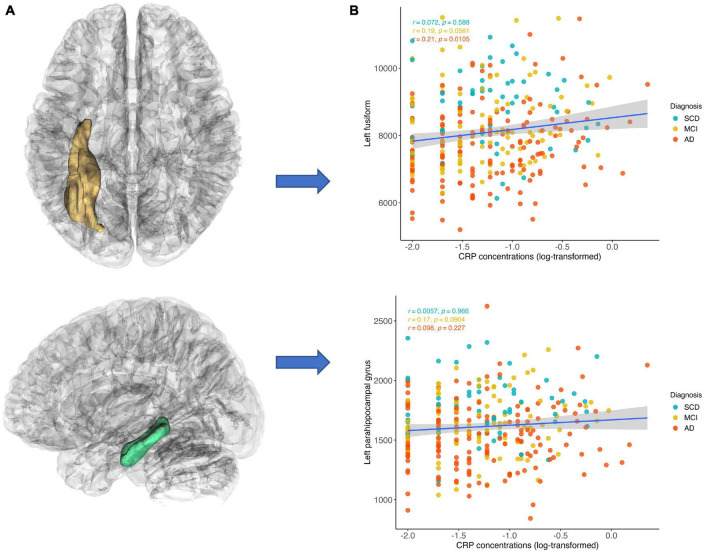
Correlations between CRP concentration and gray matter volumes. CRP level was inversely correlated with gray matter volume in the left fusiform and left parahippocampal gyrus. The positive correlation between CRP level and left fusiform were mainly driven by patients with MCI and AD groups (MCI: *r* = 0.19, *p* = 0.056; AD: *r* = 0.21, *p* = 0.011). Similarly, the positive correlation between CRP level and left parahippocampal gyrus were mainly driven by patients with MCI and AD (MCI: *r* = 0.17, *p* = 0.090; AD: *r* = 0.10, *p* = 0.227). **(A)** Region of interest and **(B)** scatter plots of correlations.

Elevated hs-CRP level was significantly associated with increased GMVs in four regions, including the left fusiform (puncorrected = 0.001, β = 0.16, 95% CI = [0.065, 0.192]), left parahippocampal gyrus (puncorrected = 0.002, β = 0.16, 95% CI = [0.057, 0.260]), right parahippocampal gyrus (puncorrected = 0.035, β = 0.11, 95% CI = [0.007, 0.208]), and left hippocampus (puncorrected = 0.018, β = 0.10, 95% CI = [0.018, 0.181]). The left fusiform (pFDR = 0.023) and left parahippocampal gyrus (pFDR = 0.029) remained significant after FDR correction (Table 2 and Supplementary Table 1). The *post hoc* analysis revealed that the significant simple correlation between CRP level and left fusiform were mainly driven by patients with MCI and AD groups (MCI: *r* = 0.19, *p* = 0.056; AD: *r* = 0.21, *p* = 0.011). Similarly, despite it did not reach the significant level, the correlation between CRP level and left parahippocampal gyrus were mainly driven by patients with MCI and AD (MCI: *r* = 0.17, *p* = 0.090; AD: *r* = 0.10, *p* = 0.227) (Figure 3). Additionally, we conducted *post hoc* analysis by using GMVs/eTIV as outcome variables to illustrate to relationship between GMVs/eTIV and CRP concentration in each group. We found that a significant positive correlation remained between CRP concentration and GMVs/eTIV in the left fusiform in the AD group (*r* = 0.20, *p* = 0.0126) (Supplementary Figure 5).

**TABLE 2:** Regional effect of hs-CRP concentration on selected ROIs.

ROI	β	SE	95% CI	*p*	p_FDR_
L caudal anterior cingulate cortex	−0.024	0.062	−0.147∼0.098	0.693	0.733
L caudal middle frontal gyrus	0.030	0.051	−0.069∼0.129	0.552	0.733
L entorhinal cortex	0.091	0.052	−0.011∼0.192	0.083	0.397
L fusiform	0.158	0.048	0.065∼0.251	0.001	0.023
L isthmus cingulate cortex	0.055	0.050	−0.044∼0.154	0.275	0.710
L parahippocampal gyrus	0.158	0.052	0.057∼0.26	0.002	0.029
L posterior cingulate cortex	−0.019	0.049	−0.116∼0.078	0.700	0.733
L precuneus	0.064	0.043	−0.021∼0.15	0.137	0.547
L rostral anterior cingulate cortex	0.036	0.055	−0.071∼0.143	0.507	0.733
L rostral middle frontal gyrus	0.040	0.040	−0.038∼0.119	0.314	0.710
L supramarginal gyrus	0.043	0.047	−0.048∼0.134	0.355	0.710
R caudal anterior cingulate cortex	0.056	0.056	−0.055∼0.166	0.327	0.710
R caudal middle frontal gyrus	−0.051	0.049	−0.146∼0.045	0.303	0.710
R entorhinal cortex	0.040	0.052	−0.062∼0.142	0.443	0.733
R fusiform	0.058	0.043	−0.027∼0.143	0.177	0.608
R isthmus cingulate cortex	−0.017	0.051	−0.118∼0.083	0.733	0.733
R parahippocampal gyrus	0.108	0.051	0.007∼0.208	0.035	0.211
R posterior cingulate cortex	−0.043	0.051	−0.143∼0.057	0.406	0.733
R precuneus	0.027	0.043	−0.058∼0.112	0.531	0.733
R rostral anterior cingulate cortex	0.020	0.054	−0.086∼0.125	0.712	0.733
R rostral middle frontal gyrus	0.023	0.041	−0.056∼0.103	0.568	0.733
R supramarginal gyrus	0.019	0.049	−0.077∼0.115	0.696	0.733
L Hippocampus	0.099	0.042	0.018∼0.181	0.018	0.142
R Hippocampus	0.016	0.045	−0.073∼0.104	0.727	0.733

Robust linear regression was conducted with CRP concentration as the independent variable and selected ROIs as outcome variables after controlled for age, sex, MMSE-J score, body mass index, drinking habits, and estimate total intracranial volume. ROI: region of interest; R: right; L: left; β: Standardized beta coefficient; 95% CI: 95% confidence interval.

### 3.3. Cognitive function and hs-CRP

**FIGURE 4:**
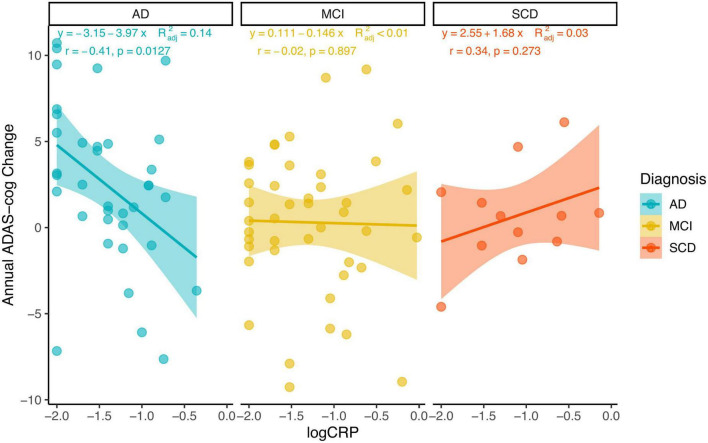
Using scatterplots with a linear regression line with 95% confidence interval and adjusted R squared, the Pearson coefficient of correlation, and its *p*-value to illustrate the relationship between hs-CRP concentration (log-transformed) and annual ADAS-cog-J change in each group.

Multivariable linear regression models were implemented to investigate the association between hs-CRP and cognitive function using unadjusted and adjusted models. Model 1 was unadjusted for covariates. Model 2 was adjusted for age, sex, body mass index, education years, and diagnosis. Model 3 was model 2 plus adjustment for the interaction between hs-CRP and diagnosis. Hs-CRP was not associated with baseline MMSE-J scores or baseline ADAS-cog-J scores in unadjusted or adjusted models. Notably, the interaction of diagnosis and hs-CRP was positively associated with annual ADAS-cog-J change (β = 0.43, SE = 1.25, *p* = 0.008) (Table 3 and Supplementary Table 2). Then, we performed Pearson correlation to test the relationship between hs-CRP concentration and cognitive function in SCD, MCI, and AD groups, respectively. Because of the limited sample size in each group, we additionally included simple linear regression equations with adjusted r squared (Figure 4). There was a negative correlation between hs-CRP concentration and annual ADAS-cog-J change in the AD group (*r* = −0.41, *p* = 0.0127). While the MCI group show no correlation and the SCD group showed a positive trend between hs-CRP concentration and annual ADAS-cog-J change (MCI: *r* = −0.02, *p* = 0.897; SCD: *r* = 0.34, *p* = 0.273).

**TABLE 3:** Multivariable linear regression analyses of the interaction effect between diagnosis and hs-CRP on cognitive function.

	Model 1		Model 2		Model 3	
	β (SE)	*p*	β (SE)	*p*	β (SE)	*p*
**Baseline MMSE-J scores**						
Hs-CRP	−0.04 (0.52)	0.509	−0.01 (0.39)	0.687	−0.07 (1.24)	0.593
Diagnosis × hs-CRP interaction					0.02 (0.50)	0.668
**Baseline ADAS-cog-J scores**						
Hs-CRP	0.00 (0.883)	0.994	−0.05 (0.72)	0.263	0.02 (2.34)	0.891
Diagnosis × hs-CRP interaction					−0.03 (0.93)	0.618
**Annual ADAS-cog-J change**						
Hs-CRP	−0.16 (0.82)	0.131	−0.10 (0.97)	0.441	0.83 (2.86)	0.024
Diagnosis × hs-CRP interaction					0.43 (1.25)	0.008

β: Standardized beta coefficient; SE = Standard error; MMSE-J = Japanese version of Mini-Mental State Examination. ADAS-cog-J: Alzheimer’s Disease Assessment Scale-Cognitive subscale Japanese version; Annual ADAS-cog-J change: (follow-up ADAS-cog-J score - baseline ADAS-cog-J score)/follow-up times (year), with higher positive annual ADAS-cog-J change score indicates greater cognitive decline; Hs-CRP: High-sensitivity C-reactive protein (log-transformed); SCD: Subjective cognitive decline, MCI: Mild cognitive impairment, AD: Alzheimer’s disease. Model 1: unadjusted association. Model 2: adjusted for age, sex, body mass index, education years, and diagnosis. Model 3: model 2 plus adjustment for the interaction between hs-CRP and diagnosis.

## 4. Discussion

This study aimed to investigate hs-CRP concentration, brain structural alterations, and cognitive function in the context of AD. We observed a significantly positive association between CRP concentration and GMVs in the left fusiform and left parahippocampal gyrus. We also investigated whether hs-CRP was associated with cognitive function. We observed a significant interaction between diagnosis and CRP concentration in predicting the annual cognitive changes; *post hoc* analysis revealed that among these patients diagnosed with AD, lower baseline hs-CRP was correlated with greater future cognitive decline. Collectively, our findings suggest that increased hs-CRP may have exert protective effect on brain structure alterations and future cognitive changes among patients already with cognitive impairment.

Elevated hs-CRP concentration was associated with increased GMVs in the left fusiform and left parahippocampal gyrus. Bettcher et al. (2012) reported that compared with undetectable group, participants with detectable CRP level (>3 mg/dL) showed smaller GMVs in the medial temporal lobe. It is noteworthy that these participants were non-demented and only the medial temporal lobe was selected. In present study, we selected 24 brain regions which has been previously linked to progression of AD and systemic inflammation. Additionally, the *post-hoc* tests revealed that the positive association between CRP level and GMVs was mainly driven by patients with cognitive impairment, that is, patients with MCI or patients diagnosed with AD. The fusiform, known as “the face area,” is critical for face perception (Kanwisher et al., 1997). Impaired facial recognition, e.g., fail to identify family members, is a common symptom in patients with AD (Hargrave et al., 2002). Amyloid deposition in the hippocampus has been linked to decreased GMVs in fusiform gyrus, suggesting an upstream and downstream relationship between these two areas (Chang et al., 2016). As part of the medial temporal lobe, the parahippocampal gyrus is mainly involved in memory processing (Köhler et al., 1998), which is a useful biomarker in neuropathology of AD (Echávarri et al., 2011; van Velzen et al., 2020). Notably, brain atrophy in the parahippocampal gyrus has been proposed to be a better predictor than hippocampus in the very early stages of AD (Echávarri et al., 2011). Our results raise the possibility that increased hs-CRP concentration may exert neuroprotective effect on brain structure alterations among patients who already with cognitive impairment, that is, increased CRP may represent an active immune function to against the progression of AD (Silverman et al., 2012). This hypothesis was proposed by Silverman et al. (2012), which they found a replicable association between increased CRP in proband and decreased dementia risk in their relatives (Silverman et al., 2012), suggesting the protective genotypes may moderate impact of systemic inflammation on AD. Supporting this notion, immune-related gene expression was found to be downregulated in subjects with cognitive impairment, while it was upregulated in the brains of cognitively healthy individuals (Katsel et al., 2009). This suggests that a robust immune system plays a central role in preserving cognitive function, whereas an inability to respond to the immune system could contribute to the disease progression (Katsel et al., 2009).

We also found a significant interaction between CRP concentration and diagnosis in predicting future cognitive decline. Specifically, increased CRP concentration at baseline was associated with slower cognitive decline in patients with AD. Our result was consistent with previous studies based on patients who already diagnosed with AD (Locascio et al., 2008; O’Bryant et al., 2010; Lima et al., 2014; Gong et al., 2016; Lewis and Knight, 2021; Saxton et al., 2023). For instance, Locascio et al. (2008) reported that a lower CRP level was associated with both greater cognitive and functional decline in patients with AD. In a recent large longitudinal study, researchers found that increased CRP was associated with worse baseline cognitive function in non-demented participants; while among participants with dementia, elevated CRP was associated with slower cognitive decline (Lewis and Knight, 2021), suggesting the impact of CRP on cognition may vary with the disease progression (O’Bryant et al., 2010; Gong et al., 2016; Fernandes et al., 2020). Despite more evidence is warranted to elucidate how CRP affects cognitive function in the neuropathology of AD, our result suggests that increased CRP level may indicate an appropriate immune response function among patients who already with cognitive impairment, contributing to slower cognitive decline in the AD progression. Our findings could also explain why epidemiological studies suggest the impact of increased inflammation on AD could be harmful; while the clinical treatments targeting on anti-inflammatory process have failed (Aisen, 2002; Wang et al., 2015; Ozben and Ozben, 2019), presumably due to a lack of considering for the different stage of progression. From this perspective, our results underline the importance of anti-inflammation treatments target on AD that should consider the different stages of this disease.

The mechanisms underlying the protective effect of CRP concentration on brain alterations and cognitive changes in patients with MCI and AD remain unclear. One potential explanation is that the presence of the Apolipoprotein ε4 (APOE ε4) allele, a well-studied genetic risk factor for AD, may modify the impact of CRP on AD-related neurodegeneration. Both animal and human studies have suggested APOE ε4 plays a role in the inflammatory process (Jofre-Monseny et al., 2008; Rebeck, 2017; Tzioras et al., 2019). It has been observed that individuals with APOE ε4 carriers exhibit lower CRP concentrations compared to non-APOE ε4 carriers, indicating potential variations in immune function among individuals with different APOE ε4 alleles (Haan et al., 2008; Hubacek et al., 2010; Tao et al., 2018; Tao et al., 2021; Wooten et al., 2021; Wang et al., 2022). Studies have reported the neuroprotective effect of increased CRP levels in both individuals without the APOE ε4 allele (Silverman et al., 2008; Lima et al., 2014; Tao et al., 2021) and APOE ε4 carriers (Haan et al., 2008). These findings may be attributed to differences in disease progression and demographic characteristics. Notably, elevated CRP levels have been associated with lower cortical Aβ specifically in APOE ε4 carriers, suggesting that increased CRP may reflect an adaptive function within this APOE ε4 genotype, contributing to improved plaque clearance (Hooper et al., 2018). However, it has also been reported that increased CRP levels have a detrimental effect on cognition in APOE ε4-positive individuals (Tao et al., 2021; Wooten et al., 2021). Further studies are needed to clarify whether APOE ε4 plays a mediating role in the association between systemic inflammation and the neuropathology of AD. Another potential explanation involves the activation of microglia. Specifically, an increased concentration of CRP may activate microglia, leading to a neurotrophic effect on the brain and cognition in patients with cognitive impairments. Peripheral inflammation can induce an inflammatory process in the brain through the activation of microglia (Riazi et al., 2008; Norden and Godbout, 2013). While sustained activation of microglia can have a neurotoxic effect and contribute to cognitive deficits, it is important to note that activated microglia can also be beneficial by producing neurotrophic compounds (Sawada et al., 2008). Notably, the transition from a neuroprotective effect to a neurotoxic effect of activated microglia depends on various factors, including disease progression and brain regions (Sawada et al., 2008). Although not directly linked to AD, the characteristics of primed microglia in individuals with Parkinson’s disease (PD) and dementia with Lewy bodies (DLB) suggest variations in the secretion of cytokines and neurotrophins, potentially resulting in neuroprotective effects in PD and neurotoxic effects in DLB (Imamura et al., 2005). It is worth mentioning that CRP can inhibit the production of nitric oxide, a neurotoxic substance produced by microglia (Chao et al., 1992). Further investigation is warranted to unravel the precise mechanisms underlying the interplay between increased CRP levels, microglial activation, and cognitive function in patients with AD and MCI.

Several limitations must be mentioned. First, despite we excluded possible confounding variable based on the detailed medical history, we cannot rule out the possibility that medication status contribute to the impact of CRP on different stages of AD. For example, the use of anti-inflammatory drugs, e.g., NSAID, could have impact on the CRP concentration. Second, our study was based on a limited sample size. We only included 313 clinical patients and 91 patients with longitudinal cognitive scores. Future studies could investigate systemic inflammation, brain alterations and cognitive changes with a larger sample size. Moreover, since we did not follow all patients, a selective bias should be considered in the longitudinal analysis. Last, we failed to include other inflammatory markers, such as TNF and IL-6 in this study. Although CRP is a sensitive marker that had been used in many previous studies, proinflammatory markers may accurately reflect the inflammatory status. Future research could examine the relationships between a broader range of inflammatory markers, brain alterations and cognitive changes in individuals with AD.

## Data availability statement

The original contributions presented in this study are included in this article/Supplementary material, further inquiries can be directed to the corresponding author.

## Ethics statement

Approval for present study was acquired by the Clinical Research Ethics Committee of the Tohoku University Graduate School of Medicine (approval number: 2018-1-618). Written informed consent to participate in this study was provided by the participants’ legal guardian/next of kin. Written informed consent was obtained from the individual(s) for the publication of any potentially identifiable images or data included in this article.

## Author contributions

YZ and YKT co-designed the study. YKT was responsible for scanning the subjects and supporting the image technology. YKT, TNK, TNG, MM, and YT collected the data. YZ, YL, and BT analyzed the data. YZ wrote the first draft of the manuscript. YYT supervised this study. All authors have contributed to and approved the final manuscript.

## Footnotes

1
https://surfer.nmr.mgh.harvard.edu/
