# Supplementary material for: Impact of hs-CRP concentration on brain structure alterations and cognitive trajectory in Alzheimer’s disease
Source: Front Aging Neurosci. 2023 Aug 1;15:1227325. doi: 10.3389/fnagi.2023.1227325 (PMC10427872; doi:10.3389/fnagi.2023.1227325)
Supplement: Supplementary file 1 [file Data_Sheet_1.docx]

**Supplementary Materials**

Table of Contents

[Supplementary Figure S1. Correlations between all variables in all patients (N=313) 2](#_Toc138163252)

[Supplementary Figure S2. Correlations between all variables in patients with SCD (N=59) 3](#_Toc138163253)

[Supplementary Figure S3. Correlations between all variables in patients with MCI (N=101) 4](#_Toc138163254)

[Supplementary Figure S4. Correlations between all variables in patients with AD (N=153) 5](#_Toc138163255)

[Supplementary Figure S5. Correlations between CRP concentration and GMVs/eTIV. 6](#_Toc138163256)

[Supplymentary Table S1. Regional effect of hs-CRP concentration on selected ROIs 7](#_Toc138163257)

[Supplementary Table S2. Multivariable linear regression analyses of the interaction effect between diagnostic status and CRP on cognitive function 8](#_Toc138163258)

# Supplementary Figure S1. Correlations between all variables in all patients (N=313)

# Supplementary Figure S2. Correlations between all variables in patients with SCD (N=59)

# Supplementary Figure S3. Correlations between all variables in patients with MCI (N=101)

# Supplementary Figure S4. Correlations between all variables in patients with AD (N=153)

# Supplementary Figure S5. Correlations between CRP concentration and GMVs/eTIV.

| Supplymentary Table S1. Regional effect of hs-CRP concentration on selected ROIs | | | | | |  |
| --- | --- | --- | --- | --- | --- | --- |
| ROI | 𝛽 | SE | 95%CI | p | pfdr |  |
| L caudal anterior cingulate cortex | -0.014 | 0.063 | -0.138~0.11 | 0.828 | 0.832 |  |
| L caudal middle frontal gyrus | 0.036 | 0.056 | -0.074~0.146 | 0.517 | 0.691 |  |
| L entorhinal cortex | 0.100 | 0.055 | -0.008~0.207 | 0.070 | 0.338 |  |
| L fusiform | 0.155 | 0.052 | 0.054~0.257 | 0.003 | **0.054** |  |
| L isthmus cingulate cortex | 0.058 | 0.056 | -0.052~0.168 | 0.300 | 0.690 |  |
| L parahippocampal gyrus | 0.145 | 0.051 | 0.045~0.245 | 0.005 | **0.054** |  |
| L posterior cingulate cortex | -0.015 | 0.051 | -0.116~0.086 | 0.771 | 0.832 |  |
| L precuneus | 0.079 | 0.056 | -0.03~0.188 | 0.155 | 0.621 |  |
| L rostral anterior cingulate cortex | 0.045 | 0.059 | -0.071~0.16 | 0.447 | 0.690 |  |
| L rostral middle frontal gyrus | 0.045 | 0.049 | -0.051~0.141 | 0.360 | 0.690 |  |
| L supramarginal gyrus | 0.055 | 0.055 | -0.051~0.162 | 0.309 | 0.690 |  |
| R caudal anterior cingulate cortex | 0.051 | 0.057 | -0.061~0.163 | 0.376 | 0.690 |  |
| R caudal middle frontal gyrus | -0.044 | 0.053 | -0.149~0.06 | 0.408 | 0.690 |  |
| R entorhinal cortex | 0.053 | 0.057 | -0.058~0.165 | 0.347 | 0.690 |  |
| R fusiform | 0.065 | 0.050 | -0.033~0.162 | 0.192 | 0.660 |  |
| R isthmus cingulate cortex | -0.012 | 0.056 | -0.121~0.097 | 0.832 | 0.832 |  |
| R parahippocampal gyrus | 0.093 | 0.051 | -0.007~0.194 | 0.068 | 0.338 |  |
| R posterior cingulate cortex | -0.039 | 0.051 | -0.139~0.061 | 0.443 | 0.690 |  |
| R precuneus | 0.034 | 0.053 | -0.07~0.138 | 0.518 | 0.691 |  |
| R rostral anterior cingulate cortex | 0.023 | 0.058 | -0.091~0.136 | 0.693 | 0.792 |  |
| R rostral middle frontal gyrus | 0.036 | 0.049 | -0.06~0.133 | 0.460 | 0.690 |  |
| R supramarginal gyrus | 0.026 | 0.059 | -0.091~0.142 | 0.667 | 0.792 |  |
| L Hippocampus | 0.110 | 0.046 | 0.019~0.2 | 0.017 | 0.140 |  |
| R Hippocampus | 0.024 | 0.049 | -0.073~0.12 | 0.627 | 0.792 |  |
| ***Note.*** Robust linear regression was conducted with CRP concentration as indepedent variable and selected ROIs as outcome variables after controled for age, sex, MMSE-J score, body mass index, and drinking. ROIs were calculated using region-to-eTIV ratio. ROI: region of interest; eTIV: estimate total intracranial volume. R: right; L: left; 𝛽: Standardized beta coefficient; 95%CI: 95% confidence interval. | | | | | |  |
|  |  |  |  |  |  |  |
|  |  |  |  |  |  |  |
|  |  |  |  |  |  |  |
|  |  |  |  |  |  |  |

|  |  |
| --- | --- |
|  |  |

| Supplementary Table S2. Multivariable linear regression analyses of the interaction effect between diagnostic status and CRP on cognitive function | | | | | | | | |  |
| --- | --- | --- | --- | --- | --- | --- | --- | --- | --- |
|  |  |  |  |  |  |  |  |  |  |
|  | Model 1 | |  | Model 2 | |  | Model 3 | |  |
|  | 𝛽 (SE) | *p* |  | 𝛽 (SE) | *p* |  | 𝛽 (SE) | *p* |  |
| **Baseline MMSE scores (N=313)** |  |  |  |  |  |  |  |  |  |
| logCRP | -0.04 (0.52) | 0.509 |  | -0.01(0.39) | 0.687 |  | -0.07 (1.24) | 0.593 |  |
| Age |  |  |  | -0.07 (0.02) | 0.094 |  | -0.07 (0.02) | 0.113 |  |
| Sex |  |  |  | -0.09 (0.42) | 0.042 |  | -0.09 (0.43) | 0.042 |  |
| Education years |  |  |  | 0.10 (0.08) | 0.027 |  | 0.10 (0.08) | 0.027 |  |
| BMI |  |  |  | 0.01 (0.06) | 0.889 |  | 0.01 (0.06) | 0.891 |  |
| Diagnosis |  |  |  | -0.63 (0.28) | 0.001 |  | -0.59 (0.65) | 0.001 |  |
| Diagnosis × CRP interaction |  |  |  |  |  |  | 0.02 (0.50) | 0.668 |  |
| **Baseline ADAS-cog scores (N=313)** |  |  |  |  |  |  |  |  |  |
| logCRP | 0.00 (0.883) | 0.994 |  | -0.05 (0.72) | 0.263 |  | 0.02 (2.34) | 0.891 |  |
| Age |  |  |  | 0.13 (0.04) | 0.013 |  | 0.12 (0.04) | 0.019 |  |
| Sex |  |  |  | 0.07 (0.76) | 0.145 |  | 0.07 (0.77) | 0.145 |  |
| Education years |  |  |  | -0.05 (0.15) | 0.306 |  | -0.05 (0.15) | 0.291 |  |
| BMI |  |  |  | -0.00 (0.11) | 0.968 |  | 0.00 (0.15) | 0.963 |  |
| Diagnosis |  |  |  | 0.57 (0.51) | 0.001 |  | 0.52 (1.25) | 0.001 |  |
| Diagnosis × CRP interaction |  |  |  |  |  |  | -0.03 (0.93) | 0.618 |  |
| **Annual ADAS-cog change (N=91)** |  |  |  |  |  |  |  |  |  |
| logCRP | -0.16 (0.82) | 0.131 |  | -0.10 (0.97) | 0.441 |  | 0.83 (2.86) | 0.024 |  |
| Age |  |  |  | -0.01 (0.06) | 0.925 |  | -0.05 (0.06) | 0.646 |  |
| Sex |  |  |  | 0.11 (0.93) | 0.345 |  | 0.13 (0.98) | 0.236 |  |
| Education years |  |  |  | 0.04 (0.34) | 0.734 |  | 0.04 (0.19) | 0.702 |  |
| BMI |  |  |  | -0.07 (0.14) | 0.557 |  | 0.04 (0.14) | 0.740 |  |
| Diagnosis |  |  |  | 0.15 (0.071) | 0.171 |  | -0.53 (1.75) | 0.057 |  |
| Diagnosis × CRP interaction |  |  |  |  |  |  | 0.43 (1.25) | 0.008 |  |
| ***Note:*** 𝛽: Standardized beta coefficient; SE = Standard error; MMSE = Mini-Mental State Examination. ADAS-cog: Alzheimer’s Disease Assessment Scale-Cognitive subscale Japanese version; Annual ADAS-cog change: (follow-up ADAS-cog score - baseline ADAS-cog score)/ follow-up times(year); SCD: Subjective cognitive decline, MCI: Mild cognitive impairment, AD: Alzheimer’s disease. Model 1: unadjusted association. Model 2: adjusted for age, sex, body mass index, education years, and diagnostic status. Model 3: model 2 plus adjustment for the interaction between CRP and diagnositic status. | | | | | | | | |  |
|  |  |  |  |  |  |  |  |  |  |
|  |  |  |  |  |  |  |  |  |  |
|  |  |  |  |  |  |  |  |  |  |
|  |  |  |  |  |  |  |  |  |  |
|  |  |  |  |  |  |  |  |  |  |
|  |  |  |  |  |  |  |  |  |  |
